# End-of-life care of patients with idiopathic pulmonary fibrosis

**DOI:** 10.1186/s12904-016-0158-8

**Published:** 2016-10-12

**Authors:** Kaisa Rajala, Juho T. Lehto, M. Saarinen, E. Sutinen, T. Saarto, M. Myllärniemi

**Affiliations:** 1 Helsinki University Central Hospital, Comprehensive Cancer Center and Faculty of Medicine, University of Helsinki, Cancer Center, PoBox 180, FI-00029, HUS, Helsinki, Finland; 2 TAYS Palliative Unit, Department of Oncology, Tampere University Hospital and School of Medicine, University of Tampere, Teiskontie 35, 33520 Tampere, Finland; 3 University of Helsinki, Faculty of Medicine, Helsinki, Finland; 4 Transplantantation Laboratory, Pulmonary Medicine, B411 Haartmaninkatu 3, FI-00290 Helsinki, Finland; 5 Department of Pulmonary Medicine, University of Helsinki and Helsinki University Hospital, Heart and Lung Center, PoBox372, FI-00029 HUS Helsinki, Finland; 6 Palliative Unit, Helsinki University Central Hospital, Comprehensive Cancer Center, Haartmaninkatu 4, FI-00290 Helsinki, Finland

**Keywords:** Idiopathic pulmonary fibrosis, Symptoms, End-of-life care, End-of-life decision, Advance care planning

## Abstract

**Background:**

Idiopathic pulmonary fibrosis (IPF) is a progressive disease with median survival from 2 to 7 years. Palliative care is an important part of patients´ care as lung transplantation is not an option for the majority of patients. The aim of this study was to describe treatment practices, decision-making and symptoms during end-of-life care of IPF patients.

**Methods:**

We identified 59 deceased patients from a national prospective IPF cohort study (FinnishIPF) and analyzed retrospectively their health care documentation during the 6 months that preceded death.

**Results:**

Hospital was the place of death for 47 patients (80 %). A majority of the patients (93 %) were hospitalized for a mean of 30 days (range 1–96 days) during the last 6 months of their life. Altogether, patients spent 15 % of their last 6 months of life in a hospital. End-of-life decisions and do not resuscitate (DNR) orders were made for 19 (32 %) and 34 (57 %) of the patients, respectively, and 22 (42 %) of these decisions were made ≤ 3 days prior to death.

During the final hospital stay, antibiotics were given to 79 % and non-invasive ventilation to 36 % of patients. During the last 24 h of life, radiologic imaging or laboratory tests were taken in 19 % and 53 % of the hospitalized patients, respectively. These tests and life prolonging therapies were more common in tertiary hospitals compared to other places of death. Dyspnea (66 %) and pain (31 %) were the most common symptoms recorded. Opioids were prescribed to 71 % of the patients during the last week before death.

**Conclusions:**

The majority of IPF patients died in a hospital with ongoing life-prolonging procedures until death. The frequent use of opioids is an indicator of an intention to relieve symptoms, but end-of-life decisions were still made very late. Early integrated palliative care with advance care plan could improve the end-of-life care of dying IPF patients.

## Background

Idiopathic pulmonary fibrosis (IPF) is a severe, progressive, chronic disease of unknown cause, seen primarily in older adults [1]. It is the most common interstitial lung disorder worldwide with a prevalence of 8.6/100 000 inhabitants in Finland according to a recent study [2].

Although there are some advances in the pharmacological treatment of IPF and some patients undergo lung transplantation, IPF is still a disease with high mortality and morbidity [3, 4]. The median overall survival of IPF patients varies from 2 to 7 years in different studies, which is comparable to many malignant disorders [4, 5]. According to a recent interview study the diagnosis of IPF has a major impact on the daily living and family relationships [6]. The symptom burden is heavy, with shortness of breath and cough being the most common symptoms [6]. Therefore, current guidelines recommend early-integrated palliative care to IPF patients in addition to early referrals to lung transplantation and pharmacological treatment to decrease lung function decline [7, 8].

Despite of the recommendations of integrated palliative care there are only a few studies on current end-of-life (EOL) care practices in patients with advanced IPF. Many IPF patients die at hospitals, but the contents of the care during the final days of life is largely unknown [9].

The aim of this study was to investigate EOL care of IPF patients with special emphasis on symptoms, current treatment practices, end-of-life decisions and the use of medical services.

## Methods

### Study population

All patients recruited to the FinnishIPF study and deceased until February 2014 were included to this study. FinnishIPF is a prospectively collected, national clinical registry study of patients with IPF diagnosed according to the ATS/ERS 2011/2015 criteria [7]. Patient recruitment was initiated in 2012 and there were 257 patients in the registry in February 2014. A detailed description of the FinnishIPF study has been published earlier [2].

### Data collection

In Finland, the majority of IPF patients are diagnosed and treated in the public sector. Electronic patient record databases and a comprehensive death registry enable access to medical data of the patients. The Finnish public health care consists of primary care with health centers and general wards, and of specialized care with secondary and tertiary hospitals. In this study, the term community hospital refers to a primary care hospital ward in local health center and tertiary hospital is defined as central or university hospital with specialized care and emergency services.

Deceased patients were identified from the FinnishIPF registry and death certificates were acquired from “National Authority for Collecting and Compiling Statistics on various fields of Society and Economy”. We collected the data concerning the patients last 6 months of life from medical records of tertiary and community hospitals including outpatient visits and records of home care. In addition, documents from private and occupational health care were evaluated if their use were mentioned inpatient records from public health care. In order to describe patients EOL care, all the given pharmacological and non-pharmacological treatments, reported symptoms as well as radiological and laboratory tests taken were recorded during the final hospital stay or the last week before death of those who died at home. The presence of symptoms was retrospectively collected from medical records. Standardized tools for symptom scores were not used in clinical practice.

Collected sociodemographic and disease characteristics included: age, sex, date of birth, date of IPF diagnosis, date and place of death, co-morbidities and smoking status. We also collected data of all visits to emergency room and hospital stays during the last 6 months of life.

Do-not-resuscitate (DNR) orders, EOL decisions, palliative care specialist consultations and referrals to hospice were recorded. In the present study we have used the term “EOL decision” for recorded decisions to start comfort only EOL care or symptom centered palliative care.

### Statistics

Characteristics of the patients were reported with descriptive statistics including means, medians, SDs, and ranges. *X* ^2^ –test was used for comparison of categorical variables between different patient groups. Two-sided *P*-value of less than 0.05 was accepted as statistically significant. Data analysis was done using SPSS version 22.

## Results

**Fig. 1:**
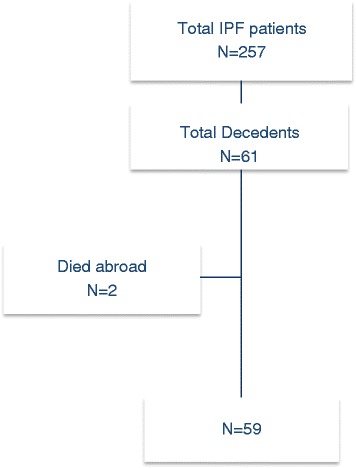
A flow-chart of the study-population

At the end of the observation period (February 2014) 61 patients identified from the FinnishIPF study had died. Two patients died abroad and were excluded due to insufficient data for analysis. Thus, 59 deceased patients formed the final study population (Fig. 1).

Patient characteristics are shown in Table 1. Eight patients (14 %) died at home, three (5 %) in a nursing home and one (2 %) in a hospice, whereas 47 (80 %) died at a hospital (three out of them in intensive care unit (ICU)). A majority of the patients (93 %) were hospitalized for a mean of 30 days (range 1–96 days) during the last 6 months of their life. Altogether, patients spent 15 % of their last 6 months of life in a hospital.

**Table 1:** Patient characteristics

	No. (%)
Patients	59
Male	32 (54 %)
Age, mean (range)	77 (56–91)
Years from diagnose to death, mean (SD)	3,7 (2,9)
Smoking	
Never	26 (44 %)
Former	28 (48 %)
Current	2 (3 %)
Missing data	3 (5 %)
Comorbidities	
Cardiovascular disease	40 (68 %)
Hypertension	28 (48 %)
Diabetes mellitus	17 (29 %)
Atrial fibrillation	13 (22 %)
Obstructive lung disease	12 (20 %)
Cancer	10 (17 %)
Comorbidities per patient	
None	4 (7 %)
1–2	21 (36 %)
3–4	29 (49 %)
> 5	5 (9 %)
Place of death	
Tertiary hospital (ward)	23 (39 %)
Tertiary hospital (ICU)	3 (5 %)
Community hospital	21 (36 %)
Home	8 (14 %)
Hospice	1 (2 %)
Nursing home	3 (5 %)
Cause of death recorded in death certificates	
IPF	42 (71 %)
Cardiovascular diseases	7 (12 %)
Cancer	6 (10 %)
Other	4 (7 %)
Hospital admissions during the last 6 months of life, median (range)	
Hospital ward visits	2 (0–6)
Length of stay per visit (days)	11 (1–48)
Length of final stay (days)	9 (1–77)

Most patients (93 %) had comorbidities, and 58 % had three or more diseases other than IPF. The cause of death was IPF in the majority of patients. Ten patients had a diagnosis of cancer (four cases of lung cancer) and cancer was the primary cause of death in six patients.

### DNR-orders and end-of-life decisions

**Fig. 2:**
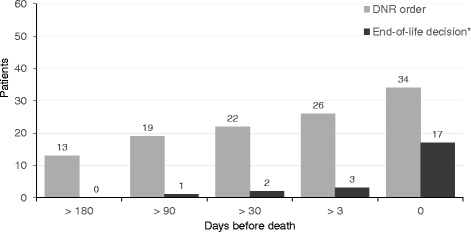
Cumulative number of DNR orders and end-of-life decisions before death in IPF-patients (*n* = 59)

Frequencies and timing of DNR-orders and EOL decisions are presented in Fig. 2. DNR order and EOL decision occurred in 34 (57 %) and 19 (32 %) of the patients, respectively. Fourteen (74 %) of the EOL decisions and 8 (24 %) of the DNR orders were made ≤ 3 days prior to death. Six (32 %) EOL decisions were made during the final 24 h of life. Fourteen (24 %) patients had both a DNR-order and an EOL decision. In 20 patients (44 %) no documented DNR order nor EOL decision was found. We didn’t find any records of palliative care specialist consultations in any of the patients and only one patient was referred to a hospice.

### Symptoms and treatments during the last week of life

Symptoms and symptom relieving treatments during the last week of life are shown in Table 2. The most common symptoms were dyspnea (66 %) and pain (31 %). Opioids were prescribed to 71 % of the patients, of which 64 % were on demand short-acting opioids and 36 % long-acting ones. Antidepressants had been prescribed for 25 % of the patients.

**Table 2:** Reported symptoms and symptom relieving medication during the last 7 days of life

Symptoms	Number (%)
Breathlessness	39 (66 %)
Pain	18 (31 %)
Delirium	11 (19 %)
Anxiety/depression	10 (17 %)
Cough	9 (15 %)
Nausea	4 (7 %)
Constipation	1 (2 %)
Medication	
Opioids	42 (71 %)
Anxiolytics	26 (44 %)

Life prolonging treatments during the last week of life and diagnostic tests during the last day of life in different care settings are presented in Table 3. Seven patients (12 %) were resuscitated. Three patients died in an ICU and two patients were intubated. Most patients (81 %) received supplemental oxygen, and 17 (29 %) were treated with non-invasive ventilation (NIV). Life prolonging treatments and diagnostic tests were common during the last days of life in patients dying at hospitals. These procedures were more frequent in tertiary hospitals compared to community hospitals (Table 3).

**Table 3:** Life prolonging treatments and diagnostic tests during the last week of life

	Place of death			
	Tertiary hospital	Community hospital	Home, nursing home or hospice	Total
Number of patients	26	21	12	59
Life prolonging treatments (last 7 days)				
NIV	13 (50 %)	4 (19 %)*	0	17 (29 %)
Parenteral fluids	16 (62 %)	11 (52 %)	2 (16 %)	29 (49 %)
Antibiotics	24 (92 %)	13 (62 %)*	2 (16 %)	39 (66 %)
Diagnostic tests (last 24 h)				
X-rays	8 (31 %)	1 (5 %)*	0	9 (15 %)
Laboratory tests	18 (69 %)	7 (33 %)*	0	25 (42 %)

*NIV* non-invasive ventilation

* *p* < 0.05 for tertiary vs. community hospital

## Discussion

In the present retrospective study we have described the end of life care of IPF patients. Our results indicate that DNR orders and end of life decisions were made late in the patients life span and life-prolonging therapies were likely to continue until the last days of life.

Due to the unpredictable disease trajectory with some patients remaining relatively stable, whereas others show a rapid lung function decline and/or acute exacerbations, discussions about EOL care preferences and early referral to palliative care are recommended [7]. In this perspective, the current guidelines seem to be poorly implemented to clinical practice. In a recent register study [9], end-of-life decisions were commonly made late in the disease course of IPF and only 14 % of the patients received referral to palliative care. Even among patients with very severe oxygen-dependent interstitial lung diseases (ILD), only 41 % of the patients underwent EOL discussions [10]. In general, patients suffering from non-malignant lung disease receive less palliative care compared to lung cancer even though the symptom burden is high in both [10, 11]. This is highlighted in a recent study by Ahmadi and co-workers who showed that 19 % of lung cancer patients receive palliative care team consultation, whereas only 6 % of ILD patients do so [10]. Only about one third of the patients, in our study had documented EOL decision and most of them were made during the last three days of life. No palliative care consultations were found and referrals to hospice were very rare, although this may partly reflect the rarity of these services in Finland. In any case, these findings from our and previous studies highlight the need for advanced care plans for IPF-patients. Our results also indicate, that DNR orders do not affect treatment practices during the final phase of IPF.

Our patients spent about 15 % of their last 6 months of life in a hospital and vast majority (80 %) of them also died there. Roughly half of the IPF-patients are reported to die in a hospital [9, 10], but our numbers are even higher. In addition, hospital admissions during the final months of life exceeded those reported in cancer [12]. The most probable reason for this result is the lack of advance care plans and limited use of palliative care services and home hospice care for non-malignant diseases. Nevertheless, these findings do not comply with patients’ wishes as most of the patients with life-limiting diseases, including IPF, prefer to be treated and die at home or a hospice [13]. Advanced care planning and arrangement of palliative home care have shown to reduce emergency room visits, hospital stays and dying in hospital setting among patients with multiple end-stage diseases [14]. Similar benefits could be achieved by early-integrated palliative care in IPF.

To our knowledge, this is the first study to describe EOL treatment practices in a registry-based population of IPF-patients. We found procedures intended to prolong life (e.g. laboratory tests, NIV and prescribing antibiotics) to be relatively common during the final days, although symptoms were treated (e.g. prescribing opioids) at the same time. This dual approach to a dying IPF-patient was probably due to the difficulty to differentiate exacerbation, secondary infection and a dying patient, but – again - also due to a late EOL decision.

In the present population one third of the patients received NIV treatment during the last week of life. NIV may relieve dyspnea as a palliative treatment, but in acute care, it is mostly used to improve survival in chronic obstructive pulmonary disease exacerbation [15–17]. The benefit of the use of NIV the symptomatic therapy of IPF patients has not been proven and, therefore, NIV is not routinely recommended [18]. Although it is understandable that NIV is used in a hope for cure or in order to relieve breathlessness, using a mask may increase and prolong suffering of the patient and prevent communication with closest ones. Therefore, the pros and cons of NIV in patients with end-stage IPF should be carefully considered. In contrast to NIV, oxygen therapy is recommended to IPF patients with hypoxemia [1]. Thus, it’s not surprising that majority of our patients received oxygen.

A significant number of radiologic and laboratory tests were ordered during the last 24 h of life and antibiotics were commonly prescribed near death. Death related to IPF is typically respiratory failure related to either progression of the disease or acute exacerbation. The clinical picture of acute exacerbation is not easily distinguished from bacterial pneumonia (elevation of c-reactive protein and pulmonary infiltrates) [19, 20]. Thus, treatment attempts with bacterial antibiotics found in our study are understandable and frequent antibiotic use is also common in patients with COPD and lung cancer during the final days of life [21]. However, the benefit of antimicrobial therapies and ordering multiple diagnostic tests should be reconsidered if the presumable prognosis of the patient is very poor (e.g. when the patient is bed-bound and highly dependent on oxygen therapy). In our population, 66 % of the patients received antibiotics during last week of life. Although these factors are not necessary indicators of poor EOL care, they reflect life-prolonging nature of the treatment close to death.

In our study, shortness of breath (66 %) and pain (31 %) were the two most common symptoms reported. In a retrospective study of ILD, shortness of breath occurred in 93 % and chest pain in 29 % of the patients, while many other symptoms like depression and fatigue were found as well [13]. In a recent study by Ahmadi et al. breathlessness (75 %), anxiety (66 %) and pain (51 %) were the most common symptoms in a mixed population of ILD patients [10]. The difference in the incidences of these symptoms may be due the retrospective nature of our study. The point of gravity in clinical patient handling may not be in the symptom-reporting, if compared to ie. questionnaire studies of clinical trials. Standardized symptom scores were not –unfortunately – a part of the evaluation of our patients. This is an important issue that should maybe be addressed in future guidelines on patient follow-up. As in other advanced lung diseases, breathlessness is obviously the main symptom in IPF. In contrast, the cause and nature of pain in IPF is unknown. This is beyond the scope of our study, but should be evaluated in future studies. Of interest, cough was reported in only 15 % of our patients, which is less than in previous studies [13]. We suggest that cough was either not very severe symptom in dying IPF-patients or health care professionals did not record the symptom.

In the present study, opioids were more frequently used than in a previous study [13]. No controlled trials support the use of opioids for shortness of breath in IPF, but there is relatively good evidence about their benefit in refractory dyspnea in general [18, 22, 23]. Therefore, the common use of opioids probably reflects a great need to control breathlessness in the dying IPF-patient. In addition, pain relief could be another reason for prescribing opioids, since nearly one-third of our patients suffered from pain. In a Swedish study anxiety was more common (66 %) in ILD patients compared to our study (17 %), but still a significant proportion of our patients had received antidepressants (25 %) and anxiolytics (44 %) [10] We suggest that psychological symptoms were not systematically recorded in patient charts, although they probably existed.

Our study is limited by a relatively small number of patients. Other limitations are the lack of more detailed information about the nature of the EOL decisions and the lack of systematic collection of patient-reported symptoms. The strength of the study is that the results represent a real-life IPF population, as most of the patient charts were identifiable from national registries.

## Conclusions

The majority of IPF patients die in a hospital setting. Death seems to be unpredictable in many cases. The late EOL decisions and the aggressive nature of the treatment during the final days reflect an unforeseeable approaching death and unplanned palliative care. Advance care plans and early EOL discussions could improve the palliative care of IPF patients.

## Abbreviations

DNR: Do not resuscitate
EOL: End-of-life
ICU: Intensive care unit
ILD: Interstinal lung disease
IPF: Idiopathic pulmonary fibrosis
NIV: Non-invasive ventilation
